# The evolution of quality in healthcare: a diffusion of innovation perspective

**DOI:** 10.1108/JHOM-05-2025-0271

**Published:** 2026-10-08

**Authors:** Mohamed Abdelsalam Osman, Shafiq Alam, Kasuni Weerasinghe, Nazim Taskin, David Pauleen

**Affiliations:** School of Management and Marketing, Massey Business School, Massey University, Auckland, New Zealand; Faculty of Business, Economics and Law, Auckland University of Technology, Auckland, New Zealand; Waikato School of Management, The University of Waikato, Hamilton, New Zealand

**Keywords:** Critical review, Health care, Quality, Diffusion of innovation

## Abstract

**Purpose:**

This article reviews the history of “quality” in healthcare through the diffusion of innovation theoretical framework, focusing on quality as an innovation within this framework.

**Design/methodology/approach:**

This article uses a qualitative critical review typology to review five key historical healthcare quality initiatives through Rogers' diffusion of innovation theoretical framework.

**Findings:**

A qualitative critical review of quality in healthcare from a diffusion of innovation lens reveals three key insights: (1) engaging opinion leaders who oppose an innovation can improve implementation; (2) developing a brand for an innovation can accelerate its diffusion rates and (3) a planned diffusion approach is crucial for maximising public health impact.

**Research limitations/implications:**

This qualitative critical review offers valuable insights; however, it is not an exhaustive review of the literature, and these outcomes could benefit from future research to support its findings and validate them across diverse healthcare settings.

**Practical implications:**

The findings suggest the need to: (1) engage diverse perspectives to refine and enhance quality initiatives. (2) Develop a branding strategy to improve the diffusion of quality initiatives. (3) Create a system-level plan that considers the broader implications for healthcare systems.

**Originality/value:**

This article provides practical guidance for healthcare professionals and policymakers, helping to improve the quality management of healthcare services. It introduces branding as a potential fifth key pillar, emphasising the involvement of opposing opinion leaders and the importance of planning in the diffusion process.

## 1. Introduction

Quality in healthcare is not a new concept, having evolved over the centuries. It dates back to the time of Hippocrates and aligns with the roots of medicine itself (Walton and Kerridge, 2007). Initially, healthcare quality focused on individual practices with little emphasis on broader systemic improvements. As healthcare systems expanded, quality improvement efforts became more organised, guided by frameworks such as Donabedian's model for assessing healthcare quality (Donabedian, 1988). At the same time, theories like diffusion of innovation (DOI) offered insights into how innovations spread within social systems, helping to explain the adoption of new healthcare practices.

Historically, approaches to quality improvement in healthcare have varied widely. In the early 20th century, the focus was primarily on standardisation and efficiency, driven by the principles of scientific management. Later, the focus shifted to patient safety and the prevention of medical errors, motivated by influential reports such as “To Err Is Human” (Institute of Medicine, 2000). More recently, there has been an increasing emphasis on patient-centred care and engaging patients in the quality improvement process (Coulter and Ellins, 2007). This evolution reflects an enhanced understanding not only of how to improve the quality of healthcare but also of what health means and how to achieve it.

There are numerous definitions of health, but the one that is widely recognised and currently in use is the definition provided by the World Health Organization (WHO), established in 1948. The WHO defines health as “a state of complete physical, mental, and social well-being, and not merely the absence of disease and infirmity” (World Health Organization, 1948). The advancement of quality in healthcare depends on embracing innovations and eliminating outdated practices to achieve that state of health. According to Gabriel Trade, one of the founding fathers of sociology, the interest in how innovations are diffused among communities can be traced back as far as 500 BCE. He inspired many researchers in the field of innovation and is considered the father of research in innovation diffusion (Kinnunen, 1996).

Everett Rogers was inspired by Gabriel Trade's research in innovation diffusion and developed the DOI theory in 1962 (Greenhalgh *et al.*, 2004). Rogers' DOI theory explains how new ideas, practices, technologies or innovations spread within social systems. Many researchers have used Rogers' DOI theoretical framework to investigate various quality innovations. For example, in healthcare, the DOI was used to study the adoption of electronic health records and Big Data analytics (Balas and Chapman, 2018; Greenhalgh *et al.*, 2004).

The motivation for this study is driven by the ongoing challenges in achieving quality in healthcare, particularly with the current technological advancements and evolving patient needs. Accordingly, understanding how quality innovations can be effectively integrated into practice becomes increasingly important.

This article aims to apply Rogers' DOI theory in a qualitative critical review of five selected historical quality innovations in healthcare. The article begins with the theoretical foundation, moving to the methodology, then analyses each of the five quality initiatives, and concludes with conclusions, implications and limitations.

## 2. Theoretical foundation: diffusion of innovation theory

The DOI is “the process by which an innovation is communicated through certain channels over time among the members of a social system” (Rogers, 2003, p. 5). This definition reflects the four main pillars of the DOI: (1) innovation, (2) communication channels, (3) time and (4) social system (Rogers, 2003).

Innovation is the first pillar, and is viewed as a new idea, practice or object whose characteristics would either facilitate or hinder its diffusion rate. These characteristics include relative advantage, compatibility, complexity, trialability and observability (Rogers, 2003). Communication channels, the second pillar, are vital for spreading innovation throughout the diffusion process. The third pillar, time, is an integral factor in determining the rate of diffusion of any innovation, whilst the fourth and last pillar is the social system, which he defined as “interrelated units that join together to solve a problem or accomplish a goal” (Rogers, 2003, p. 24). Identifying these individuals in the social system showed that they have different roles based on their behaviours and characteristics (Straub, 2009; Greenhalgh *et al.*, 2004). These roles include opinion leaders, who influence others' opinions about innovation (Rogers, 2003; Greenhalgh *et al.*, 2004).

Individuals who adopt an innovation are categorised into five groups based on how quickly they adopt new ideas. Innovators are the first to adopt, followed by early adopters who help gain widespread acceptance. The early majority adopts next, as they see the benefits, followed by the late majority, who are more reluctant, and lastly, the laggards, who resist change (Rogers, 2003).

We reviewed our selected theory against two commonly used theories in the DOIs: the Unified Theory of Acceptance and Use of Technology (UTAUT) (Venkatesh *et al.*, 2003) and the Technology Acceptance Model (TAM) (Davis, 1989). The four pillars of UTAUT include performance expectancy, effort expectancy, social influence and facilitating conditions, which determine technology use (Venkatesh *et al.*, 2003). In contrast, TAM focuses on perceived usefulness and perceived ease of use as the key drivers of technology adoption (Davis, 1989). Although UTAUT and TAM provide important perspectives on technology adoption, their emphasis on technology and individual factors limits their relevance to innovations in healthcare quality. As seen in the comparison of frameworks (see Appendix), DOI appears to be a more suitable theoretical lens for examining the historical diffusion of quality innovations in healthcare.

DOI provides a valuable framework for understanding how innovations diffuse. However, critics have identified several limitations, including DOI's tendency to focus on adoption at the individual level, with less attention to system-level influences that can shape diffusion (Greenhalgh *et al.*, 2004). Additionally, the framework has been criticised for treating diffusion as a largely linear process and for paying insufficient attention to the influence of power, resistance and competing interests on adoption decisions (Fitzgerald *et al.*, 2002). These limitations are particularly relevant in healthcare, where the adoption of innovation often occurs within complex organisational and social environments rather than through individual choice alone. As a result, factors such as trust, credibility, legitimacy and reputation may influence diffusion alongside the traditional DOI pillars.

Building on these four pillars, this review proposes branding as a fifth pillar of the DOI framework. Branding is the strategic building of relationships between consumers and products, services and organisations (Evans *et al.*, 2015). The value of branding lies in its ability to influence how target audiences respond to an innovation by developing awareness and positive associations (Keller, 1993). This influence is equally evident in healthcare, where brands shape engagement and adoption of services, interventions and health-related behaviours (Kemp *et al.*, 2014). Branding strengthens the diffusion process by helping innovations gain trust, credibility and legitimacy within their social system through alignment with the values and expectations of potential adopters (Suchman, 1995). In the context of DOI theory, branding is defined as the strategic development of a relationship between the target audience and the innovation through communication channels over time by creating a distinct identity for the innovation that aligns with the expectations and values of the social system. Unlike the other DOI pillars, which focus on the technical aspects of diffusion, branding directly influences the success and rate of diffusion by shaping how an innovation is perceived, trusted and legitimised. It integrates with the four pillars by enhancing the innovation through a clear identity and urgency, ensuring communication resonates with the target audience, influencing the time required to reach early and late majorities, and aligning the innovation with the social system's values to support broader acceptance. The following review examines how this fifth pillar operated across five historical quality innovations.

## 3. Methodology

This article aims to explain the historical advancements in quality within healthcare through a qualitative critical review guided by the DOI theoretical framework. For the purposes of this review, “quality” refers to the degree to which healthcare services increase the likelihood of desired health outcomes and are consistent with current professional knowledge (Institute of Medicine, 1990). An “innovation” is defined as a new idea, practice or object (Rogers, 2003). A “quality innovation” in healthcare, therefore, refers to a novel approach or technology implemented to enhance healthcare services (Rogers, 2003). This article connects historical quality innovations with the pillars of DOI by applying DOI theory, illustrating the journey of how these innovations have been adopted over time.

The research follows Paré *et al.* (2015) qualitative critical review typology, which aligns with the research objectives. Previous studies have employed a qualitative review approach to assess healthcare and evaluate interventions (Paré *et al.*, 2015). This critical review is selective rather than exhaustive, emphasising the identification of key strengths, weaknesses and inconsistencies in the current knowledge of selected quality innovations. This method facilitates a deeper examination of the chosen innovations and their connection to DOI theory. Unlike a systematic review, this review does not aim to compile all available evidence; instead, it offers a targeted examination of significant historical milestones (Paré *et al.*, 2015).

In keeping with this approach, sources relating to each innovation were identified purposively, drawing on key historical and healthcare quality literature and supplemented through snowball sampling of seminal works, with priority given to primary historical analyses and well-established secondary accounts (Paré *et al.*, 2015). Each of the five innovations was then examined using the four DOI pillars as the analytical framework. For each case, the evidence was assessed against innovation attributes, communication channels, time and social system, while the presence and role of branding were evaluated using the definition established in the theoretical foundation. This common analytical approach was applied consistently across all five cases to support comparability while allowing the distinctive features of each historical context to emerge. Given the interpretive and theory-generative aims of the review, the analysis seeks to identify recurring patterns and insights rather than provide an exhaustive historical account or statistically generalisable findings.

Table 1 presents a purposeful selection of five quality innovations, following the chronological structure suggested by Sheingold and Hahn (2014) for reviewing the historical evolution. Five innovations were chosen to balance diverse healthcare milestones with deep analysis. This approach enables a comprehensive examination, focusing on a manageable number of sources for thorough analysis (Paré *et al.*, 2015).

**Table 1:** Quality innovation selection criteria

Quality innovation	Year	Quality evolution milestone	Lasting impact on today's healthcare
Hygienic measures	1840	First quality documentation	Remains a fundamental aspect of infection control
Germ theory	1860	Acceptance of the role of microorganisms in causing infectious diseases	The prevailing scientific understanding of numerous diseases
Standardisation and accreditation of healthcare	1911	Ensures consistent quality of care, patient safety and accountability in healthcare delivery	The current quality of healthcare relies on accreditation and standardisation criteria
Penicillin discovery	1928	Transformed the treatment of bacterial infections, significantly reducing mortality and morbidity associated with infectious diseases	Antibiotics, as bacterial killers, are considered the greatest medical breakthrough of the 20th century
Industrial quality model use in healthcare	1996	Application of industrial quality management principles to healthcare	Industrial quality models' impact on quality in healthcare is still being researched

**Source(s):** Authors’ own work

The selection criteria were developed to ensure that the chosen innovations were relevant to the research aim and could provide meaningful insights into the application of DOI theory. The criteria include: (1) A key event in the history of healthcare: The innovation represents an advancement in healthcare practices (Sheingold and Hahn, 2014). (2) The impact of innovation remains evident in today's quality of healthcare. (3) It reflects at least one of the four pillars in the DOI in quality in healthcare (Rogers, 2003).

## 4. Findings

### 4.1 Hygienic measures: same approach, different outcomes

#### 4.1.1 Background

In the early 19th century, resources for treating illnesses were limited, and physicians mostly focused on classifying diseases. Healthcare quality relied on individual practitioners' self-regulatory efforts to improve standards. According to Rogers (2003), innovations often start by addressing a problem. However, recognising quality issues in healthcare was rare then, as questioning it seemed inappropriate due to the belief that being a doctor ensured high standards. These norms allowed medical practitioners to avoid implications of malpractice. There was little pressure from patients, family or external factors on practitioners to enhance healthcare quality.

#### 4.1.2 The innovation

The 19th century experienced rising mortality rates in hospitals and wars, prompting healthcare innovations. One significant development was Dr Ignaz Semmelweis's work in the 1840s, which linked handwashing to puerperal fever and maternal death. Handwashing and other hygienic practices clearly innovated healthcare services. His findings corresponded with the DOI framework, showing clear benefits, compatibility and ease of diffusion with positive results (Sheingold and Hahn, 2014). However, his innovation met resistance as it implicated doctors in causing harm, suggesting professional failure. His strict adherence to hospital protocols, without sufficient consultation, alienated the medical community. Furthermore, his emotional and confrontational writings impeded acceptance (Paul *et al.*, 2024).

In the 1850s, Florence Nightingale was inspired by Semmelweis's hygienic measures. During the Crimean War, she applied these principles, successfully reducing mortality among British troops. Unlike Semmelweis, Nightingale documented her findings with statistics and visuals, communicating effectively with military leaders and policymakers. Her reforms were framed as life-saving measures for soldiers, securing broad institutional support (McDonald, 2010).

#### 4.1.3 Insights from hygienic measures

Dr Semmelweis and Florence Nightingale both advocated for hygiene, documentation and cleanliness, yet they achieved different outcomes due to differing approaches to their messaging and audience engagement (Hines *et al.*, 2020; Sheingold and Hahn, 2014).

Dr Semmelweis targeted pregnant women in hospitals. However, he positioned the opinion leaders within the social system, doctors and obstetricians, as responsible for maternal deaths, leading to their resistance to his innovation due to their professional pride (Paul *et al.*, 2024). Consequently, the diffusion of his innovation was limited. Furthermore, he faced demotion and criticism from opinion leaders despite evidence supporting his findings (McDonald, 2010).

Nightingale's focus was on winning the war and reducing mortality among soldiers, which helped increase acceptance of the innovation. However, the important factor that contributed to Nightingale's success was her ability to convince opinion leaders to support her by framing the approach in a patriotic narrative of saving soldiers to win the war, which aligned with the opinion leaders, the military and political leaders (McDonald, 2010).

Rogers' (2003) analysis emphasised the role of opinion leaders in the social system, influencing the DOI. While these leaders could support or oppose innovations, as seen with Dr Semmelweis, this was not the case for Florence Nightingale. Throughout her journey, she faced criticism and attacks, as noted in writings such as “Florence Nightingale: Reputation and Power” (Bostridge, 2008). Despite this opposition, she successfully diffused her innovation (McDonald, 2010) by effectively branding it and attracting key opinion leaders to support her. For example, Nightingale gained support from prime ministers and cabinet members for reforming War Office procedures (McDonald, 2010). Her success in branding this innovation was framed within the DOI theoretical framework, facilitating effective communication and enhancing acceptance within the healthcare system. This highlights the importance of framing messaging to promote innovative practices.

### 4.2 Germ theory: the invisible innovation

#### 4.2.1 Background

The first industrial revolution contributed to the transformation of quality in healthcare. It began in 1765 with the invention of steam engines and the establishment of steam-powered factories. The growth of many industries in the early 19th century, including textile and iron, attracted more people to work in the cities. However, the increasing crowding in small areas with poor housing and sewage systems resulted in the spread of various diseases, such as typhoid, cholera and smallpox (Roberts, 2015; Ajmera and Jain, 2019). As these diseases spread, many physicians were innovators in trying to limit their spread.

#### 4.2.2 The innovation

Among the notable physicians who contributed to limiting the spread of these diseases is the British physician John Snow, who made one of the early breakthroughs. In 1854, he was able to trace the source of a cholera outbreak to the infamous Broad Street water pump and thus eliminated access to the contaminated water. His contribution laid the groundwork for further explorations that contributed to the discovery of disease-causing microorganisms, paving the way for Louis Pasteur's “Germ Theory” in 1862 and the emergence of bacteriology (Berger, 1999).

Research followed the innovation, leading to widespread acceptance of germ theory. Robert Koch, a German physician, established that specific germs cause specific diseases by extracting and cultivating them. This increased the DOI, and opinion leaders embraced Pasteur and Koch's work. In the 1870s, scientists like Thomas Huxley and John Tyndall further validated germ theory by demonstrating bacteria's developmental phases.

The establishment of germ theory revolutionised healthcare, shifting societal perception. This shift facilitated the early diffusion of quality approaches linked to medical advancements, like the stethoscope and ophthalmoscope, and refined diagnostic methods such as temperature, heart rate and blood pressure (Berger, 1999; Beck and Melo, 2014). The late 19th century saw significant advancements, including the discovery of vaccines such as the rabies vaccine in 1885 and the diphtheria vaccine in 1891.

#### 4.2.3 Germ theory from a DOI perspective

Germ Theory, a transformative concept in healthcare, qualifies as an innovation. It introduced a new understanding that changed medical practices and public health. Unlike hygienic measures, germ theory lacked attributes for diffusion. At the time, microorganisms causing diseases were not visible, and their role was unproven. Thus, germ theory took about 20 years to become established in the 1880s when research confirmed innovation attributes like observability (Yu, 1966).

Rogers (2003, p. 250) characterised those who are last to adopt innovation as “laggards”, describing them as isolated and with “almost no opinion leadership”. Contrary to this, the diffusion of germ theory did not align with the laggards' characteristics. Florence Nightingale, an innovator and opinion leader, initially rejected germ theory and only adopted it in 1884. Even after embracing it, her scepticism can be observed in her statement addressing nurses in 1897: “A great doctor, a friend of mine, says, ‘Call it germs, *bacillus* or dirt, the treatment is the same, that is, cleanliness’” (McDonald, 2010, p. 8). This indicates that being an opinion leader, innovator or laggard is not a universal trait but depends on context, allowing one to be an innovator in some cases and a laggard in others.

Opinion leaders play an integral role in the diffusion rate of innovations (Rogers, 2003). Their support is considered to increase the diffusion rate, while their opposition is thought to slow it down. However, our findings show some variance. In the case of germ theory, opposition from opinion leaders strengthened the innovation itself. Charleton Bastian, a physiologist and strong supporter of “Archebiosis”, the belief in the spontaneous generation of living matter, opposed Pasteur's work and rejected germ theory until his death. However, his opposition was largely well-founded, forcing Pasteur to refine his methods and improve innovation (Richmond, 1954). This highlights that opposition does not necessarily hinder an innovation. It can contribute to its success by promoting further improvement and reinforcing its foundation when it effectively meets several key pillars of the DOI, such as relative advantage, compatibility and observability.

The example of the Germ theory follows the DOI theory, which links the rate of diffusion with the innovation's attributes and the social system. Initially resisted due to the invisibility of microorganisms and conflicting medical beliefs, its acceptance increased with scientific validation. Early communication lacked observable evidence, slowing adoption. Over time, accumulating evidence enhanced the theory's attributes, specifically observability, leading to its widespread adoption in the healthcare social system.

This analysis, using the DOI theory, highlights key findings: an innovation's success relies not only on its scientific validity but also on the opposition it faces, which encourages improvements and enhances its attributes. The gradual acceptance of germ theory over time allowed for the strengthening of innovation attributes, such as evidence and observability. As communication within the social system evolved, healthcare quality came to focus on reducing mortality through sanitary measures in hospitals and communities to prevent the spread of germs (Zivaljevic *et al.*, 2016).

### 4.3 Standardising quality in healthcare

#### 4.3.1 Background

Healthcare facility standardisation and accreditation began in the 20th century. In 1911, surgeon Ernest Amory Codman from Massachusetts was inspired by Taylor's management principles and Henry Ford's methods in automobile factories (Locke, 1982; Best and Neuhauser, 2006) to explore how industrial management could enhance healthcare quality, advocating for standardisation and outcome-based systems. He implemented end-result systems, error classification and patient record-keeping, viewed as early evidence-based medicine (Biscontini, 2024). In 1917, he motivated 300 professionals and hospital administrators from the American College of Surgeons to create an accreditation process establishing minimum operational standards for hospitals (Berg, 2004; Hines *et al.*, 2020).

In 1928, the American College of Surgeons established the Association of Record Librarians of North America (ARLNA) to standardise methods. By 1952, collaboration among physicians and surgeons formed the Joint Commission on Accreditation of Hospitals (JCAH), making accreditation a requirement for healthcare facilities. This non-profit organisation maintains minimum hospital standards and operates today as “The Joint Commission”, recognised as the leading organisation in healthcare quality improvement in the United States and internationally.

#### 4.3.2 The innovation

The innovation is the standardising and accrediting of healthcare practices. Initially, it was slowly adopted as early adopters like Codman and ARLNA promoted the concept. Further adoption increased as societal views on healthcare began to shift. The initial slow spread resulted from a lack of alignment with the existing healthcare system.

As the healthcare system evolved, people began to live longer, which led to a rise in non-communicable diseases such as heart disease and diabetes. This change, along with a shift in how we view health, encouraged standardisation in healthcare practices. Notably, the WHO's 1948 redefinition of health brought attention to the importance of physical, mental and social well-being, highlighting the need to address both infectious and chronic diseases to enhance the overall quality of healthcare.

In his 1966 paper, Avedis Donabedian outlined three critical domains for evaluating quality in healthcare: structure, process and outcome. This foundational work laid the basis for assessing healthcare quality, which was further enhanced by contributions from early adopters and the Joint Commission.

#### 4.3.3 Standardisation and accreditation in context: a DOI perspective

The diffusion of healthcare standardisation parallels hand hygiene and germ theory. Florence Nightingale branded her innovation to align with her era's values. In contrast, Henry Ford applied scientific management to manufacturing, but this did not immediately benefit healthcare. Branding strategies are not universally applicable across social contexts. Early healthcare adopters were influenced by Ford's methods, but it was only when the broader social system prioritised long-term outcomes that healthcare accepted and integrated standardisation.

Healthcare standardisation, like germ theory, experienced slow diffusion due to initial rejection by social systems. The main challenge was that these innovations clashed with established social values. Germ theory required additional research and observable evidence to align with the social system. In contrast, healthcare standardisation prompted a shift in the social system's understanding of health, focusing on infectious diseases and chronic conditions, thus aligning with healthcare standards.

The gradual, incremental or steady DOI can be more effective, as seen with germ theory and healthcare standardisation. In the case of germ theory, this gradual spread allowed the innovation to strengthen, while for healthcare standardisation, it enabled the societal system to evolve its perception of health. This emphasises that the interaction between the social system, innovation and time is crucial for successful diffusion. Rogers (2003) noted that high rates of diffusion are often seen positively, while slower rates are viewed negatively. However, these healthcare innovations show that a slower pace can yield substantial benefits, allowing the innovation to better align with changing societal perceptions regarding health.

### 4.4 Antibiotics in providing healthcare

#### 4.4.1 Background

The Second Industrial Revolution, which began in 1870, led to major technological and social advancements (Roberts, 2015; Sarfraz *et al.*, 2021). According to Rogers (2003), one of the drivers of innovation is the consequences of prior innovations. The industrial advancements of this period resulted in larger populations concentrated in factories and cities, leading to an increase in communicable diseases, such as influenza and tuberculosis, due to overcrowded conditions.

#### 4.4.2 The innovation

Researchers shifted focus to communicable diseases, leading to one of the 20th century's crucial discoveries, Penicillin, by Nobel Prize winner Alexander Fleming (Sheingold and Hahn, 2014). Despite its potential for treating communicable diseases, penicillin was largely ignored for over a decade as society prioritised industrial progress and factory workers over medical advancements. The focus remained on economic growth and workforce health rather than on investing in long-term treatments for infectious diseases.

In 1939, the Second World War escalated, prompting the British Government and the Rockefeller Foundation to fund Dr Florey at Oxford University to further research on Fleming's work (Ligon, 2004). The U.S. entry into the war in 1941 sparked renewed interest in penicillin, leading to large-scale research on its extraction and production. By 1944, mass production had become sufficient to treat soldiers on the battlefield (Ligon, 2004; Barr and Podolsky, 2020).

Following the war, penicillin was branded as a “wonder drug” and became a cultural icon. Branding efforts were heavily influenced by opinion leaders from medicine, politics and science, increasing their rate of diffusion beyond early adopters to the early and late majorities (Strasser, 2008). This branding strategy played a key role in shifting public perception and ensuring the widespread use of penicillin.

#### 4.4.3 Key learnings from antibiotics in providing healthcare

The diffusion of penicillin closely mirrored Nightingale's advocacy for hand hygiene, particularly in the way both innovations were driven by war, government support and strategic framing. Just as Nightingale's implementation of hygienic measures during the Crimean War was critical in improving survival rates and eventually evolving medical practice, the wartime need for penicillin accelerated its diffusion. In both cases, governments played a key role in facilitating mass adoption, not necessarily due to a genuine belief in the science behind the innovations but because they were framed as critical tools for winning wars (McDonald, 2010). This framing created an urgent need that shifted both social and political perceptions, allowing for rapid diffusion in ways that would not have been possible under other social systems.

After the war, penicillin, despite being the same innovation, was reintroduced into the same social system with the same medical needs, but framed differently. It became a “Wonder drug”, heavily branded and endorsed by opinion leaders who shaped public perception (Strasser, 2008). The DOI theory explains the impact of social systems on innovation, yet its bell-shaped curve fails to clarify the 10-year gap between early adopters and the early majority of penicillin's diffusion. Moore (2014) identified this gap as the “chasm”, where innovations struggle to transition from early adopters to the early majority. Penicillin's wartime investment provided a push, but post-war branding ensured its continued diffusion. Moore (2014) argued that strong investment is necessary for innovations to cross this chasm. In penicillin's case, branding functioned as a distinct force alongside the four established pillars of DOI: social system, communication channels, innovation and time, suggesting it is a critical but overlooked factor in healthcare innovation diffusion.

In discussions on germ theory and healthcare standardisation, we highlighted how slow DOI can be beneficial, countering Rogers' (2003) view that high diffusion rates are always positive. Penicillin, while saving lives, illustrates how rapid diffusion, driven by branding as a “wonder drug”, can lead to unintended consequences. Its impulsive adoption resulted in overuse, contributing to antibiotic resistance and shifting physicians primarily as antibiotic prescribers rather than holistic providers (Strasser, 2008). This emphasises the need for a planned perspective on diffusion rates in healthcare, where controlled diffusion may be more beneficial than rapid, unregulated DOI.

The case of penicillin aligns with Balas and Chapman's (2018) critique of DOI theory, particularly their discussion on “impulsive adoption”. They argue that some innovations spread rapidly due to strong branding rather than thorough evaluation, paralleling the post-war branding of penicillin as a wonder drug. The acceptance of innovations based on perception rather than long-term assessment highlights a limitation in DOI theory, especially in healthcare, where unregulated diffusion can have severe consequences.

### 4.5 Industrial quality models diffusion to healthcare

#### 4.5.1 Background

The evolution of quality in industries, especially in the early 20th century, significantly influenced current healthcare quality approaches. Innovators such as Walter Shewhart, William Deming and Joseph Juran initiated the quality movement, highlighting structured frameworks like Statistical Process Control (SPC) and the Plan-Do-Study-Act (PDSA) cycle, as well as Juran's Quality Trilogy, which comprises planning, control and improvement (Best and Neuhauser, 2006). These methods introduced a systematic, data-driven approach to enhance quality, initially transforming manufacturing by improving efficiency and reducing waste. However, adapting these industrial-quality models for healthcare presented challenges. Healthcare features a complex social system involving diverse stakeholders, each facing unique challenges in balancing patient needs, professional concerns, population health and efficiency. This complexity led to ongoing debate about the applicability of industrial quality frameworks that prioritise patient outcomes and safety over production efficiency (Macrae and Stewart, 2019).

By the late 20th century, adapted quality frameworks began gaining acceptance in global healthcare systems, including the Malcolm Baldrige National Quality Award (MBNQA), the European Foundation for Quality Management (EFQM) and the Finnish Quality Award (FQA), all aimed at enhancing healthcare outcomes through structured management practices similar to those in industry (John, 2000). Most healthcare organisations implement quality management systems to meet established standards (Bandyopadhyay and Coppens, 2005).

#### 4.5.2 The innovation

The adaptation of industrial quality models to healthcare aims to enhance patient care and safety. The demand for improved healthcare has prompted organisations to use these frameworks to systematically improve outcomes and address social system pressures (Carman *et al.*, 2010).

Integrating industrial quality models into healthcare required careful alignment with the healthcare social system's values and needs. This process needed modifications to the models, adjustments to the healthcare framework and shifts in stakeholders' perspectives on quality management. It involved branding the models for the healthcare community, highlighting their benefits over current practices and demonstrating their potential to improve patient outcomes and organisational performance (Carman *et al.*, 2010).

#### 4.5.3 DOI analysis of industrial quality models diffusion to healthcare

Analysing the adoption of industrial quality models in healthcare through DOI reveals parallels with earlier healthcare innovations. Like Henry Ford's mass production in healthcare, embracing industrial quality models also needed a transformation (Van den Heuvel, 2007). This involved training professionals in quality management and creating organisational cultures focused on continuous improvement (Carman *et al.*, 2010). Another similarity is how the germ theory posed challenges for the healthcare social system. Similarly, implementing industrial quality models demands adjustments to existing healthcare structures (Macrae and Stewart, 2019). Effective framing was crucial for integrating industrial quality models, as with hygiene practices and antibiotics.

The social system was crucial in spreading industrial quality models in healthcare. The “To Err is Human” report revealed high medical error rates, pressuring the healthcare system (Institute of Medicine, 2000). It highlighted the need for systematic approaches to reduce harm and improve care, aligning with quality management principles and supporting the integration of industrial quality models into healthcare (Van den Heuvel, 2007). Healthcare leaders branded these models as tools to enhance patient safety, reduce errors and boost efficiency (Carman *et al.*, 2010). This branding was crucial for acceptance, similar to how penicillin was marketed as the “wonder drug” (Best and Neuhauser, 2006). Positioning industrial quality models as essential for improving healthcare outcomes was strategic. This branding was key for adoption, highlighting their importance for evolving healthcare needs (John, 2000). Branding tends to be an important pillar in the diffusion process, alongside the traditional four pillars of DOI (social system, communication channels, innovation and time).

In this case, the branding of industrial quality models was reinforced through the endorsement and support of recognised organisations such as the MBNQA, the EFQM and the Joint Commission. These organisations provided the credibility and legitimacy, positioning these models as compatible with healthcare's goals of improving quality, safety and patient outcomes (Suchman, 1995). Unlike penicillin, which was widely recognised as the “wonder drug”, or Nightingale's reforms, which were strongly associated with her personal reputation and leadership, industrial quality models gained acceptance through the reputation and influence of the institutions promoting them. Although the form of branding differed, its role in the diffusion process remained similar by establishing a clear identity for the innovation, building trust among potential adopters and supporting its acceptance within the healthcare system.

The diffusion of industrial-quality models in healthcare parallels earlier innovations like germ theory and penicillin, which required effective framing and branding for adoption. Healthcare-specific quality frameworks, such as MBNQA and EFQM, aimed to adapt industrial models to healthcare's unique needs (Van den Heuvel, 2007).

### 4.6 Summary of findings

This analysis reveals new insights, specifically the role of branding in improving the diffusion process. Branding shapes views, creates emotional connections and ensures innovations align with societal values, thereby increasing their adoption. Table 2 summarises how these innovations align with the DOI pillars and the emerging significance of branding as a potential fifth pillar. The key findings are outlined below:

**Table 2:** Summary of quality innovations in healthcare: DOI pillars and branding role

Quality innovation	Key DOI pillars demonstrated	Branding approach	Diffusion outcome
Hygienic measures	Innovation attributes (Relative Advantage), Communication Channels, Social System	Personal Reputation: Nightingale's branding of innovation, reputation and public image enhanced the adoption	Failed under Semmelweis and succeeded under Nightingale (opinion leaders engaged)
Germ theory	Innovation attributes (Relative Advantage, Compatibility, Complexity)	Scientific branding: relying on research and publications	Slow diffusion initially then widespread; opposition (Bastian) forced refinement and strengthened observability
Standardisation and accreditation	Innovation attributes (Compatibility), Communication Channels, Social System	Institutional Support: branding through the creation of accreditation bodies (e.g. Joint Commission)	Gradual adoption; accelerated once the social system values (long-term outcomes) aligned with innovation
Penicillin discovery	Innovation attributes (Relative Advantage, Trialability, Observability)	Cultural Association: the “Wonder drug” branding accelerated adoption	Rapid diffusion; branding-accelerated, which later led to unintended consequences (overuse, resistance)
Industrial quality models	Innovation attributes (Compatibility, Complexity, Trialability)	Institutional Support: branding of methodologies (e.g. the European Foundation for Quality Management (EFQM))	Gradual diffusion; Initial resistance due to incompatibility, then institutional endorsement led to wide acceptance

**Source(s):** Authors’ own work

1. *Role of* *opposing* *opinion* *leaders:* Leaders who oppose innovations can contribute positively by encouraging rigorous evaluation and refinement, thus improving the innovation and increasing its chances of success.
2. *Branding as a* *pillar in DOI:* Branding plays a critical role in accelerating the diffusion process. It shapes perceptions, creates emotional connections and ensures innovations align with societal values, thereby enhancing their adoption.
3. *Planned* *diffusion* *approach:* A measured, gradual diffusion approach ensures that innovations are thoroughly evaluated, particularly in the context of public health, before branding strategies are applied for broader adoption.

## 5. Discussion

This study analysed five healthcare innovations through the lens of Rogers' DOI theory: hygienic measures, germ theory, quality standardisation, antibiotics and industrial quality models. We explored their integration into healthcare by considering innovation, communication channels, timing and social systems. Our results corresponded with these foundations, providing fresh insights for future investigation.

First, we reviewed opposing leaders' roles in innovation diffusion. While DOI theory sees them as obstacles, our review suggests they can significantly enhance the innovation process. Opposition can improve evaluation and ultimately, as seen with germ theory, it overcame scepticism through constructive criticism from opinion leaders, facilitating acceptance (Fischhoff, 2020). Including opposing leaders in discussions makes the diffusion process more resilient and adaptable to diverse perspectives. Such opposing opinion leaders differ from Rogers' (2003) laggards. Laggards are late or passive adopters, whereas opposing opinion leaders hold influence and actively challenge an innovation, and this engagement can strengthen it, as Bastian's opposition did for germ theory.

Second, we found that diffusion rate does not always correlate with innovation success or failure, as Rogers suggested. While he viewed rapid diffusion as success and slow diffusion as potential failure, our review shows this framework has limitations. For example, although Penicillin diffused quickly, its rapid adoption caused challenges like antibiotic resistance due to overuse (Ventola, 2015). Thus, fast diffusion can lead to negative consequences. Conversely, slower diffusion of Germ Theory allowed for a measured approach, ultimately strengthening its methods and encouraging further research. Therefore, diffusion rate should not be a clear indicator of success or failure but is influenced by the nature of the innovation and its societal impact (Fischhoff, 2020).

Third, our review aligns with Moore's (2014) findings on the gap between early adopters and the early majority, which he referred to as the “chasm”. While our findings confirm Moore's observation, we offer a different perspective. Moore argues that government support is essential to bridge this gap, but we believe branding is the key factor that connects this divide throughout the entire diffusion process. Branding shapes perceptions and aligns innovations with the early majority's needs, enabling broader adoption. By emphasising branding as a crucial pillar, we showcase its potential to impact the diffusion process across all stages rather than relying solely on external support (Moore, 2014).

Establishing branding as a fifth pillar requires demonstrating that it contributes something the existing four pillars do not: not the mechanics of how an innovation spreads, but how it is perceived and legitimised. Three observations from this review support this proposition. First, branding is distinct from communication channels. Semmelweis and Nightingale promoted similar innovations through comparable communication channels, yet only Nightingale's strategically branded reforms achieved widespread diffusion, suggesting that branding influences trust, meaning and acceptance in ways that the transmission of information alone does not (Suchman, 1995). Second, branding appears to be more than an incidental factor, as diffusion was limited where branding was weak despite strong innovation attributes, as seen in both Semmelweis's hygienic measures and the early resistance to germ theory. Third, branding was evident across all five innovations spanning almost two centuries and markedly different social systems (Table 2). A factor that is distinct from the existing pillars, influences diffusion outcomes and is consistently evident across diverse contexts suggests a broader structural role in the diffusion process, supporting branding's position as a fifth pillar rather than a peripheral or moderating condition.

Across the five cases, despite occurring in different eras, healthcare contexts and social systems, legitimacy, trust, credibility and acceptance emerged as recurring influences on diffusion. While these factors originated from different sources, they consistently supported the adoption and spread of innovation. In Nightingale's reforms, legitimacy was closely linked to her personal reputation and public influence. Germ theory gained credibility through scientific evidence, publication and validation by the wider scientific community. Penicillin achieved widespread recognition and acceptance through its reputation as the “wonder drug”, while industrial quality models derived legitimacy from established accreditation and quality organisations such as the MBNQA, EFQM and the Joint Commission. Although the source and form of influence differed across cases, a common pattern emerged: innovations diffused more successfully when they developed a trusted and credible identity that aligned with the values and expectations of the social system. These recurring characteristics suggest the presence of branding in different forms across all five innovations. Whether derived from individuals, scientific communities, public reputation or institutions, branding contributed to diffusion by establishing legitimacy, building trust and supporting acceptance, thereby supporting its position as a fifth pillar alongside the four traditional pillars of DOI. Figure 1 presents the proposed extended DOI framework developed from this review.

**Figure 1:**
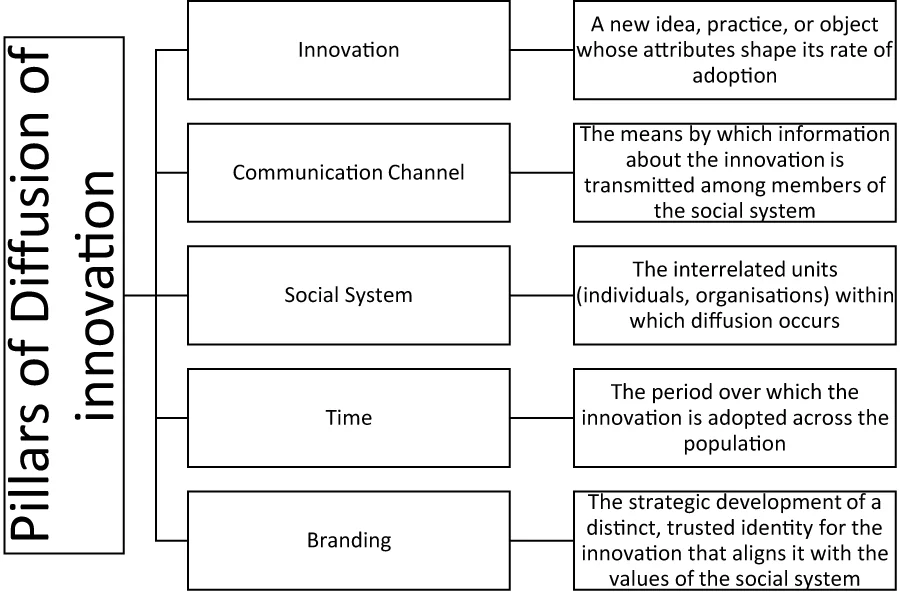
Proposed extended DOI framework with branding as a fifth pillar. Source: Authors’ own work

Rogers' DOI theory has four main pillars: innovation, communication channels, time and social systems. Our review suggests integrating branding as a fifth pillar. This addition offers a comprehensive understanding of how innovations are communicated and adopted in healthcare. Our updated DOI definition is: “Diffusion of Innovation is the process by which an innovation is strategically branded and communicated through specific channels over time among members of a social system”. This highlights branding's role in shaping perceptions and facilitating adoption in healthcare, positioning it as the fifth pillar of the DOI framework. In this way, branding directly addresses the limitations of DOI noted earlier: its limited attention to legitimacy, power and the strategic framing of innovations via introducing branding as a fifth pillar through which innovation gains acceptance within the social system (Greenhalgh *et al.*, 2004; Fitzgerald *et al.*, 2002).

## 6. Conclusion and implications

This qualitative critical review examines healthcare quality evolution through the DOI lens. Findings show that branding affects innovation adoption and diffusion by shaping perceptions and integration into healthcare systems. Branding can enhance the DOI theory by influencing innovation, societal values and stakeholder engagement. The review stresses the importance of diverse perspectives, including those resistant to change, and underscores the need for a clear diffusion strategy for successful implementation. However, qualitative critical reviews are constrained by the literature and subjective interpretations, as well as by the focused scope of five cases. As a result, the findings are intended to be theory-generative rather than broadly generalisable, and require further research, including systematic reviews, to validate them in various healthcare contexts.

### 6.1 Theoretical implications

Branding tends to influence the DOI framework by shaping how innovations are understood and valued. It defines an innovation's identity, legitimacy and perceived urgency, which can either accelerate or manage diffusion. Building on the evidence across the five cases, this review positions branding as a fifth pillar that interacts with innovation, communication, time and social systems, while further research can test and refine its operation across contemporary healthcare contexts. Additionally, DOI theory should further explore resistance dynamics and strategic diffusion planning, moving beyond just individual adoption decisions.

### 6.2 Practical implications

This review guides management and policymaking. Findings suggest: (1) Engage diverse perspectives: seek stakeholder feedback to enhance quality initiatives. (2) Develop a branding strategy to highlight innovation benefits and align with stakeholder values, facilitating adoption. (3) System-level planning: Create a diffusion plan addressing broader healthcare implications for effective implementation.

These findings have practical implications for healthcare organisations introducing new innovations. New technologies such as AI-enabled diagnostic tools, digital health platforms and cloud-based patient information systems may benefit from branding strategies that emphasise patient safety, reliability and alignment with clinical values rather than focusing solely on efficiency or cost savings (Florea, 2015; Wissawaswaengsuk *et al.*, 2025). Healthcare organisations should engage diverse stakeholders throughout the diffusion process, address concerns openly and promote education, transparency and collaboration to strengthen trust and credibility. The impact of branding strategies may be reflected through indicators such as adoption rates, time to adoption, stakeholder engagement and implementation success. However, branding must be applied responsibly. Because branding shapes perceptions and can accelerate adoption, there is a risk of misbranding, overstatement or misinformation, where acceptance is driven more by reputation than evidence. Developing clear diffusion plans that consider branding alongside innovation, communication channels, time and social system may support successful adoption while ensuring that trust and acceptance remain grounded in demonstrated benefits and outcomes.

### 6.3 Future research directions

This review explores branding's role in innovation diffusion but suggests broader implications. Future research must identify key factors affecting healthcare innovation, such as regulatory barriers, cultural influences and organisational readiness. Specifically, future studies could examine whether branding-led diffusion produces more durable adoption than diffusion driven primarily by regulatory mandate or financial incentive, and under what conditions.

Further studies should examine branding's ethical considerations, its role in various healthcare systems and its long-term effects on innovation sustainability. This could include comparative testing across different system types, such as public versus private or high-income versus lower- and middle-income contexts, to identify the factors that moderate branding's effect, as well as closer examination of the ethical boundary between strategic framing that serves patient benefit and branding that drives adoption beyond what the evidence supports.

Research should further test how branding operates as a fifth DOI pillar and how it influences healthcare innovation adoption across contexts. Additionally, strategies for managing resistance to change and implementing diffusion plans in diverse healthcare contexts should be explored. Collectively, these directions point towards the empirical validation of branding as a fifth pillar across different healthcare contexts.

## Appendix

**Table A1:** Comparison of frameworks for analysing innovation diffusion

Framework	Key concepts focus	Strengths	Weaknesses
Diffusion of innovation (DOI) (Rogers, 2003)	- Innovation characteristics	Focuses on the process of adoption over time Highlights key factors influencing adoption Useful for retrospective analysis	Innovation bias: limited focus on individual perceptions.
	- Communication channels		
	- Time		
	- Social system		
	- Branding as a fifth pillar		
Technology acceptance model (TAM) (Davis, 1989)	Perceived usefulness	Focuses on user perceptions of technology	Primarily focuses on individual-level adoption
	Perceived ease of use	Useful for predicting individual adoption	It does not consider social or organisational factors
Unified theory of acceptance and use of technology (UTAUT)	Explains user intentions and behaviour in adopting information systems by integrating eight theories into four Core constructs - Performance expectancy - Effort expectancy - Social influence - Facilitating conditions	Comprehensive; strong predictive power; considers moderating factors like age, gender and experience	Limited scope: It may not be suitable for explaining the diffusion of innovations that are not primarily technology-based Complex compared to simpler models Individual-focused

**Source(s):** Authors’ own work
